# Achieving Highly Efficient Warm‐White Light Emission in All‐Inorganic Copper‐Silver Halides via Structural Regulation

**DOI:** 10.1002/advs.202303501

**Published:** 2023-08-03

**Authors:** Sijia Wang, Runze Liu, Juntao Li, Fengke Sun, Qing Yang, Shunshun Li, Jianyong Liu, Junsheng Chen, Pengfei Cheng

**Affiliations:** ^1^ State Key Laboratory of Molecular Reaction Dynamics Dalian Institute of Chemical Physics Chinese Academy of Sciences Dalian 116023 P. R. China; ^2^ University of Chinese Academy of Sciences Beijing 100039 P. R. China; ^3^ Institute of Molecular Sciences and Engineering Institute of Frontier and Interdisciplinary Science Shandong University Qingdao 266237 P. R. China; ^4^ Key Laboratory of Chemical Lasers Dalian Institute of Chemical Physics Chinese Academy of Sciences Dalian Liaoning 116023 P. R. China; ^5^ Nano‐Science Center and Department of Chemistry University of Copenhagen Universitetsparken 5 Copenhagen DK‐2100 Denmark

**Keywords:** copper halides, scintillators, self‐trapped excitons, structural regulation, warm‐white light

## Abstract

Single‐component metal halides with white light emission are highly attractive for solid‐state lighting applications, but it is still challenging to develop all‐inorganic lead‐free metal halides with high white‐light emission efficiency. Herein, by rationally introducing silver (Ag) into zero‐dimensional (0D) Cs_3_Cu_2_Br_5_ as new structural building unit, a one‐dimensional (1D) bimetallic halide Cs_6_Cu_3_AgBr_10_ is designed that emits strong warm‐white light with an impressive photoluminescence quantum yield (PLQY) of 94.5% and excellent stability. This structural transformation lowers the conduction band minimum while maintaining the localized nature of the valence band maximum, which is crucial in expanding the excitation spectrum and obtaining efficient self‐trapped excitons (STEs) emission simultaneously. Detailed spectroscopy studies reveal that the white‐light originates from triplet STEs emission, which can be remarkably improved by weakening the strong electron‐phonon coupling and thus suppressing phonon‐induced non‐radiative processes. Moreover, the interesting temperature‐dependent emission behavior, together with self‐absorption‐free property, make Cs_6_Cu_3_AgBr_10_ as sensitive luminescent thermometer and high‐performance X‐ray scintillator, respectively. These findings demonstrate a general approach to achieving effective single‐component white‐light emitters based on lead‐free, all‐inorganic metal halides, thereby opening up a new avenue to explore their versatile applications such as lighting, temperature detection and X‐ray imaging.

## Graphical abstract

Herein, an unprecedented one‐dimensional copper‐silver halide Cs_6_Cu_3_AgBr_10_ is developed by rationally introducing Ag into zero‐dimensional Cs_3_Cu_2_Br_5_. As consequences of the structural transformation, Cs_6_Cu_3_AgBr_10_ exhibits highly efficient warm‐white light with PLQY up to 94.5%, whereas Cs_3_Cu_2_Br_5_ shows weak blue emission. This warm‐white light is ascribed to triplet self‐trapped excitons, which can be greatly enhanced by weakening the strong electron‐phonon coupling effect.

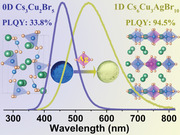

## 1 Introduction

Artificial lighting accounts for ≈20% of global electricity consumption.^[^ ^1^ ^]^ Single‐component white light emitting materials are ideal for lighting applications because they can effectively avoid the color instability, high energy consumption, and self‐absorption issues faced by commercial multi‐component white‐light sources.^[^ ^1^ ^]^ In recent years, low dimensional metal halides have emerged as a unique class of functional materials with diverse compositions and superior optical properties.^[^ ^2^, ^3^ ^]^ Large structural disorder and strong quantum confinement effect are the most attractive features of these compounds, which are expected to lead to broadband white light emission.^[^ ^4^, ^5^, ^6^ ^]^ However, the lead toxicity, organic cations’ thermal instability, and relatively low PLQYs are the major concerns hindering their applications for solid‐state lighting.^[^ ^7^ ^]^

Numerous efforts have been devoted to developing highly luminescent and stable metal halides based on lead‐free and earth‐abundant elements.^[^ ^8^, ^9^ ^]^ Among them, all‐inorganic copper(I) halides have attracted much attention due to their environmental friendliness, high stability, simple processing and efficient luminescence.^[^ ^10^, ^11^, ^12^ ^]^ Cu(I) halides are energetically stable within a 3‐ or 4‐fold coordination instead of a 6‐fold coordination,^[^ ^13^ ^]^ making them tend to form 0D or 1D structures with large exciton binding energy. The dimension mentioned here is related to the connection of metal halide polyhedral units at the molecular level instead of materials’ macroscopic size. Low dimensional all‐inorganic copper halides, such as Cs_3_Cu_2_X_5_, Rb_2_CuX_3_ and K_2_CuX_3_,^[^ ^14^, ^15^, ^16^, ^17^, ^18^ ^]^ exhibit high PLQYs with emission limited to the blue light range, which is determined by the dominant contribution of copper(I) orbitals in the electronic transition.^[^ ^19^ ^]^ In addition, all‐inorganic copper halides generally suffer from narrow excitation band (< 300 nm), which may restrict their practical applications in light‐emitting diodes.^[^ ^20^ ^]^ Therefore, it is urgent to regulate their optical properties to meet the requirements of lighting applications. Regulating the composition and structure of metal halides are two effective ways to tune their optical properties. In terms of compositional regulation, doping can improve white light efficiency by breaking parity‐forbidden transitions,^[^ ^1^, ^7^ ^]^ or expand spectral coverage by introducing emission complementary to the host metal halides.^[^ ^11^, ^21^ ^]^ However, the optical properties of metal halides are highly sensitive to trace dopants, making large‐scale production and reproducibility difficult. With regard to structural regulation, although it has profound effects on electronic structure and consequently photoluminescence (PL) properties, it is rarely reported. We believe it deserves further exploration to tailor the optical properties of metal halides to achieve highly efficient white light emission.

Herein, we design an air‐stable, 1D all‐inorganic mixed copper and silver halide Cs_6_Cu_3_AgBr_10_ by introducing Ag into 0D Cs_3_Cu_2_Br_5_ as new structural building unit. Noticeably, this structure transformation results in boosted triplet STEs emission, with emission color changing from blue to warm‐white, PLQY increasing from 33.8% to 94.5%, and excitation band shifting from 330 to 380 nm. The origin of efficient white light emission is ascribed to weakened electron‐phonon coupling and thus suppressed nonradiative recombination processes, as demonstrated by detailed spectroscopy measurements and density functional theory (DFT) calculations. By virtue of its all‐inorganic chemical composition, Cs_6_Cu_3_AgBr_10_ shows impressive stability toward heat, moisture, and light. Furthermore, it was successfully applied to white light‐emitting diode (WLED), luminescent thermometer and X‐ray imaging, exhibiting versatile application prospects.

## 2 Result and Discussion

**Figure 1:**
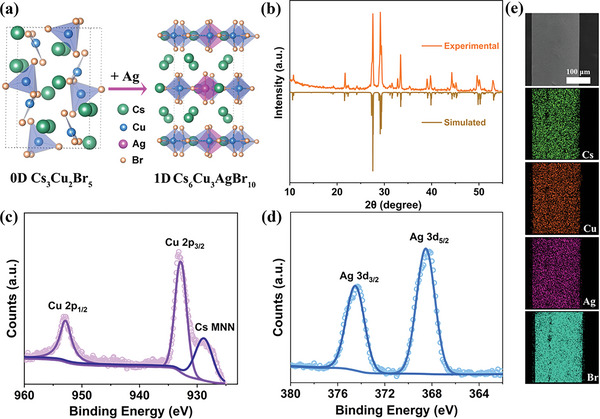
a) Schematic plot of the Ag‐incorporating‐induced structural transformation from the 0D Cs_3_Cu_2_Br_5_ to 1D Cs_6_Cu_3_AgBr_10_. b) PXRD patterns and simulated patterns from SCXRD of Cs_6_Cu_3_AgBr_10_. XPS spectra of c) copper and d) silver in Cs_6_Cu_3_AgBr_10_. e) EDS mapping of rod‐like Cs_6_Cu_3_AgBr_10_ showing the uniform distribution of Cs, Cu, Ag and Br elements.

High‐quality Cs_6_Cu_3_AgBr_10_ single crystals were prepared by the cooling crystallization method. Photographs of as‐prepared single crystals are shown in Figure S1 (Supporting Information). Single‐crystal X‐ray diffraction (SCXRD) analysis elucidates that Cs_6_Cu_3_AgBr_10_ adopts an orthorhombic space group Cmcm with a 1D structure at the molecular level, and the detailed lattice parameters are listed in Table S1 (Supporting Information). Both Cu^+^ and Ag^+^ ions coordinate with four Br^−^ ions to form tetrahedral [CuBr_4_]^3−^ and [AgBr_4_]^3−^, respectively. Two tetrahedrons are assembled into a dimer by edge‐sharing, which further forms a 1D chain structure through corner‐sharing (**Figure** **1**a). Powder X‐ray diffraction (PXRD) patterns of Cs_6_Cu_3_AgBr_10_ are in good agreement with the simulation results from SCXRD (Figure 1b), indicating the high phase purity of the as‐prepared compound. X‐ray photoelectron spectroscopy (XPS) survey confirms the presence of Cs, Cu, Ag and Br in Cs_6_Cu_3_AgBr_10_ (Figure S2, Supporting Information). The Cu 2p region exhibits two characteristic peaks with binding energies of 952.9 and 932.9 eV (Figure 1c), assigned to Cu^+^.^[^ ^22^ ^]^ Meanwhile, the peaks centered at 368.5 and 374.5 eV correspond to Ag^+^ 3d_5/2_ and 3d_3/2_, respectively.^[^ ^23^ ^]^ Moreover, the energy dispersive spectroscopy (EDS) measurement gives a Cu/Ag atomic ratio of 3.05: 1, consistent with the stoichiometry. In addition, the EDS mapping images reveal the homogeneous element distribution of Cs, Cu, Ag, and Br (Figure 1e), ruling out the existence of other phases.

**Figure 2:**
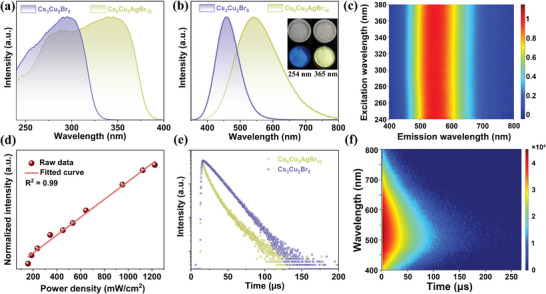
a) PLE and b) PL spectra of Cs_6_Cu_3_AgBr_10_ and Cs_3_Cu_2_Br_5_ crystals. The inset shows the photographs of Cs_3_Cu_2_Br_5_ (left) and Cs_6_Cu_3_AgBr_10_ (right) single crystals under daylight and UV light. c) Normalized excitation wavelength‐dependent PL spectra of Cs_6_Cu_3_AgBr_10_ at room temperature. d) Emission intensity of 545 nm versus excitation power for Cs_6_Cu_3_AgBr_10_. e) TRPL decay curves of Cs_6_Cu_3_AgBr_10_ and Cs_3_Cu_2_Br_5_. f) Contour plot of the TRPL spectra of Cs_6_Cu_3_AgBr_10_.

The photoluminescence excitation (PLE) spectra of both samples are shown in **Figure** **2**a, which match well with the UV‐vis absorption spectra (Figure S3, Supporting Information). The 1D Cs_6_Cu_3_AgBr_10_ shows an excitation cutoff edge at *λ* ≈ 380 nm, which is obviously red‐shifted compared to that of 0D Cs_3_Cu_2_Br_5_ at around *λ* = 330 nm. The inset of Figure 2b shows the rod‐like Cs_6_Cu_3_AgBr_10_ crystals, which are colorless and transparent under ambient light and emit extremely bright warm‐white light under 365 nm ultraviolet light. By contrast, blue‐emitting Cs_3_Cu_2_Br_5_ can only be excited at 254 nm rather than 365 nm. Cs_6_Cu_3_AgBr_10_ crystals show a broadband emission centered at 545 nm with a full‐width‐half‐maximum (FWHM) of 157 nm and a large stokes shift of 195 nm, while the Cs_3_Cu_2_Br_5_ exhibits a narrow emission located at 460 nm with a FWMH of 81 nm. Note that the PLQY is 94.5% for Cs_6_Cu_3_AgBr_10_, which is much higher than that of Cs_3_Cu_2_Br_5_ (≈33.8%) and is comparable to the highest values recently reported for metal halides with white light emission (Table S2, Supporting Information).

To further investigate the mechanism of the broadband emission, PL spectra of Cs_6_Cu_3_AgBr_10_ were measured at different excitation wavelengths (from 240 to 380 nm, Figure 2c). Despite the presence of two metal halide species, only a single PL peak centered at 545 nm is observed, indicating that only one emission center in Cs_6_Cu_3_AgBr_10_ at room temperature. In addition, the PL intensity of Cs_6_Cu_3_AgBr_10_ exhibits linear dependence on the excitation power (Figure 2d), further excluding the emission mechanism from permanent defects. The time‐resolved PL (TRPL) decay curve of Cs_6_Cu_3_AgBr_10_ gives an average lifetime of 9.8 µs, close to that of Cs_3_Cu_2_Br_5_ (Figure 2e). Such long lifetimes indicate that the corresponding emissions are likely from the spin‐forbidden triplet states.^[^ ^10^ ^]^ Moreover, pseudocolor TRPL spectra of Cs_6_Cu_3_AgBr_10_ are present in Figure 2f, which shows a ten‐microsecond‐long lifetime across the detection region (from 400 to 800 nm), indicating that the ultra‐broad emission is from the radiative relaxation process from the same excited state. The wide emission band, large Stokes shift, together with long lifetime (≈10 µs) indicates that the triplet STEs from the soft lattice of Cs_6_Cu_3_AgBr_10_ contributes to its characteristic warm‐white light emission. In this context, structural regulation may have a significant impact on the luminescence performance of these materials by adjusting their lattice structure. In order to reveal the structure‐property relationship of low dimensional metal halides, we focused on the influence of silver‐induced structural regulation on optical performance.

**Figure 3:**
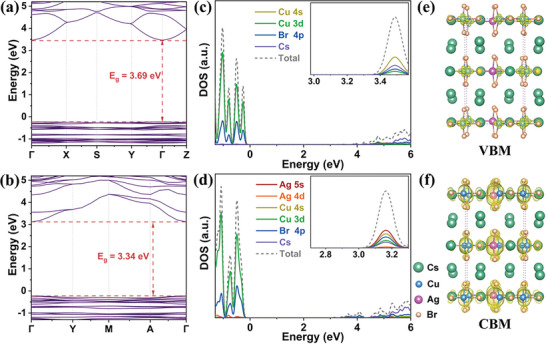
Calculated electronic band structure of a) Cs_3_Cu_2_Br_5_ and b) Cs_6_Cu_3_AgBr_10_. Projected density of states of c) Cs_3_Cu_2_Br_5_ and d) Cs_6_Cu_3_AgBr_10_. e) VBM and f) CBM‐associated charge density maps of Cs_6_Cu_3_AgBr_10_.

To reveal the impact of introducing Ag on the optical properties, DFT calculations were carried out for obtaining the electronic structures and density of states (DOSs) of Cs_3_Cu_2_Br_5_ and Cs_6_Cu_3_AgBr_10_, as shown in **Figure** **3**. It can be seen that both compounds show direct bandgaps at the *Γ* point. The bandgaps of Cs_3_Cu_2_Br_5_ and Cs_6_Cu_3_AgBr_10_ are 3.69 and 3.34 eV (Figure 3a,b), respectively, which are in good agreement with the experimental results (Figure S4, Supporting Information). The narrowing of the bandgap after introducing Ag^+^ matches well with the extended excitation spectrum of Cs_6_Cu_3_AgBr_10_ in our experimental observation. Notably, the valence bands of both compounds are flat, which is beneficial for the formation of self‐trapped holes and could attract surrounding electrons through Coulomb interaction to form STEs. After incorporating Ag into Cs_3_Cu_2_Br_5_ to form Cs_6_Cu_3_AgBr_10_, the valence band maximum (VBM) is still contributed by the Cu 3d and Br 4p orbitals, while the conduction band minimum (CBM) is derived from the Ag 5s, Cu 4s and Br 4p orbitals (Figure 3c,d). Therefore, the introduction of silver atoms mainly contributes to CBM, which can also be confirmed by the charge distribution on VBM and CBM of Cs_6_Cu_3_AgBr_10_ (Figure 3e,f). The charge density concentrated in Cu‐Br and Ag‐Br clusters is isolated by Cs^+^ ions in the 1D direction, so that excitons are confined in each bimetallic chain, which renders large exciton binding energy and strong quantum confinement effect, further enabling high photoluminescence efficiency.

**Figure 4:**
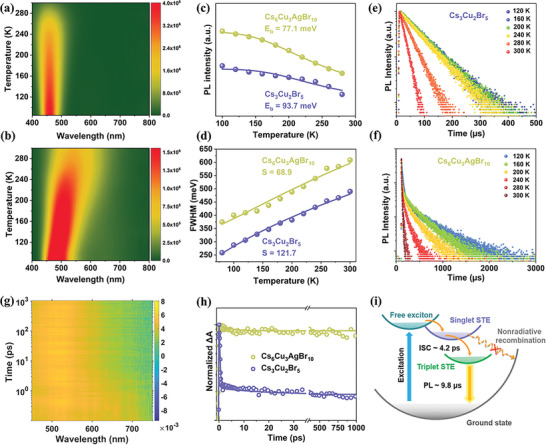
Contour plots of temperature‐dependent PL spectra of a) Cs_3_Cu_2_Br_5_ and b) Cs_6_Cu_3_AgBr_10_. c) Integrated PL intensity and d) FWHM as a function of temperature for Cs_3_Cu_2_Br_5_ and Cs_6_Cu_3_AgBr_10_. PL decay curves of e) Cs_3_Cu_2_Br_5_ and f) Cs_6_Cu_3_AgBr_10_ at different temperatures. g) Pseudocolor plot of fs‐TA spectrum of Cs_6_Cu_3_AgBr_10_ upon photoexcitation at 310 nm. h) Normalized PIA decay curves of Cs_3_Cu_2_Br_5_ and Cs_6_Cu_3_AgBr_10_ at 590 nm. i) Schematic diagram of the photophysical processes in Cs_6_Cu_3_AgBr_10_.

To gain deep insights into the impact of Ag^+^ incorporation on the STEs emission properties of Cs_6_Cu_3_AgBr_10_, we measured the temperature‐dependent PL spectra. As shown in **Figure** **4**a, the emission peak position of Cs_3_Cu_2_Br_5_ is almost independent of temperature, which is consistent with previous literature.^[^ ^24^ ^]^ In contrast, the PL spectra of Cs_6_Cu_3_AgBr_10_ show a continuous red‐shift as the temperature increases from 80 K to 300 K (Figure 4b), which may result from the shrunk bandgap out of the enhanced electron‐phonon interaction with ambient temperature raised.^[^ ^25^, ^26^ ^]^ We performed differential scanning calorimetry (DSC) measurements (Figure S5, Supporting Information) and ruled out the phase transition at the temperature range. With increasing temperature, the PL intensities of Cs_3_Cu_2_Br_5_ and Cs_6_Cu_3_AgBr_10_ decrease, which are accompanied with broadening of FWHM and shortening of lifetime (Figure 4e,f). This is likely due to the increase of thermally populated vibrational states and the enhancement of nonradiative recombination at high temperature.^[^ ^27^, ^28^ ^]^

By fitting the Arrhenius curve of temperature and integral PL intensity,^[^ ^29^ ^]^ the calculated exciton binding energy (E_b_) of Cs_6_Cu_3_AgBr_10_ is 77.1 meV (Figure 4c), which is slightly less than that of Cs_3_Cu_2_Br_5_ (93.7 meV), as a result of the increase in dimensions induced by structure transformation. This value is still much larger than the thermal activation energy (≈26 meV) at room temperature,^[^ ^30^ ^]^ indicating the formation of stable excitons in Cs_6_Cu_3_AgBr_10_. Furthermore, the Huang‐Rhys factor (*S*) used to evaluate the electron‐phonon coupling strength can be obtained from equation 1:^[^ ^31^ ^]^

(1) $$\mathrm{FWHM}=2.36\sqrt{S}\hbar \omega_{\mathrm{phonon}}\sqrt{\coth\frac{\hbar \omega_{\mathrm{phonon}}}{2k_{B}T}}$$

where *ћω*_phonon_, *k_B_*, and *T* stand for phonon energy, Boltzmann constant and temperature, respectively. As shown in Figure 4d, the *S* value is calculated to be 121.7 for Cs_3_Cu_2_Br_5_, which is significantly larger than that of Cs_6_Cu_3_AgBr_10_ (68.9). In general, large *S* values indicate strong electron‐phonon coupling, which favors STEs formation. However, an excessively large *S* value may result in the intersection of the ground state and excited state potential energy surfaces in the configuration coordinate diagram, indicating that excitons can recombine nonradiatively via releasing phonons, leading to weak PL emission. Note that the introduction of Ag reduces the electron‐phonon coupling and adjusts *S* to a moderate value, which not only ensures the formation of STEs, but also prevents the nonradiative process, contributing to the highly efficient triplet STEs emission in Cs_6_Cu_3_AgBr_10_.

We further performed femtosecond transient absorption (fs‐TA) measurements to study the ultrafast excited‐state dynamics of STEs. Under 310 nm laser excitation, the pseudo‐color fs‐TA plots of Cs_3_Cu_2_Br_5_ and Cs_6_Cu_3_AgBr_10_ both exhibit broad photo‐induced absorption (PIA) signals (Figure 4g; Figure S6, Supporting Information), which are typical features of STEs.^[^ ^32^ ^]^ The PIA dynamics show similar rise time within ≈300 fs (Figure S7 and Figure S8, Supporting Information), indicating that the STEs formation processes are almost barrierless.^[^ ^33^ ^]^ Figure 4h compares the PIA decay curves of Cs_3_Cu_2_Br_5_ and Cs_6_Cu_3_AgBr_10_ at 590 nm. The PIA decay signal of Cs_3_Cu_2_Br_5_ can be fitted with a tri‐exponential function, comprised of an ultrafast component of 0.31 ps, a middle component of 17.6 ps, and a long lifetime of far more than 1 ns (Figure 4h). After ruling out the interference of the Auger effect by adjusting the pump fluence (Figure S9, Supporting Information), the ultrafast component is assigned to phonon‐mediated decay pathways due to the overlarge electron‐phonon coupling as discussed above. The middle component is assigned to intersystem crossing (ISC) from singlet to triplet STEs, and the long lifetime corresponds to the radiative triplet STEs lifetime. For Cs_6_Cu_3_AgBr_10_, the ultrafast component is absent while the slow radiative component is dominant, which may be responsible for the highly efficient STEs emission. The fitted PIA decay lifetime of Cs_6_Cu_3_AgBr_10_ consists of a middle component of 4.2 ps and a slow process with lifetime of far more than 1 ns. Similarly, we exclude the possibility of the Auger effect (Figure S10, Supporting Information) and assign the middle component to ISC process.^[^ ^21^ ^]^ Note that the ISC process of Cs_6_Cu_3_AgBr_10_ (4.2 ps) is faster than that of Cs_3_Cu_2_Br_5_ (17.6 ps), which may be related to the stronger spin‐orbit coupling (SOC) effect caused by the introduction of heavier silver element.

Based on the above analysis of spectral measurement results, the proposed broadband white light emission mechanism of Cs_6_Cu_3_AgBr_10_ is shown in Figure 4i. Upon photoexcitation, the singlet STEs derived from free excitons form rapidly through moderate exciton‐phonon coupling interaction, avoiding phonon‐induced nonradiative recombination. Subsequently, the singlet STEs are transferred to triplet STEs by a fast ISC process (4.2 ps), eventually leading to an efficient warm‐white emission with a long lifetime (9.8 µs) and a large Stokes shift.

Apart from the attractive PL properties, Cs_6_Cu_3_AgBr_10_ shows excellent stability toward humidity, light, and heat, which are desirable for its versatile applications. After being exposed to ambient air for one year, almost no change in PXRD patterns was observed (Figure S11, Supporting Information). In addition, the PLE and PL spectra of the aged Cs_6_Cu_3_AgBr_10_ crystals remain unchanged (Figure S12, Supporting Information), further confirming its outstanding stability. Moreover, after heating at 70 °C or continuous UV irradiation for 12 h, the crystal structure of Cs_6_Cu_3_AgBr_10_ was well preserved, as confirmed by XRD patterns (Figure S11, Supporting Information). Thermogravimetric analysis (TGA) reveals that Cs_6_Cu_3_AgBr_10_ can be stable up to 847 K (Figure S13, Supporting Information). In contrast, the color of Cs_3_Cu_2_Br_5_ crystals changed from white to dark purple after one month of storage in the air (Figure S14, Supporting Information), which is due to the oxidation and formation of Cu^2+^ compound.^[^ ^34^ ^]^ To investigate the underlying mechanism of stability improvement, we compared the high‐resolution XPS spectra of Cs_3_Cu_2_Br_5_ and Cs_6_Cu_3_AgBr_10_ (Figure S15 and S16, Supporting Information). After Ag introducing, the characteristic peaks of Cu and Br shift slightly towards the higher binding energy, indicating that the chemical interactions between Cu and Br have been somewhat strengthened, which is beneficial to improve the stability of Cs_6_Cu_3_AgBr_10_.

**Figure 5:**
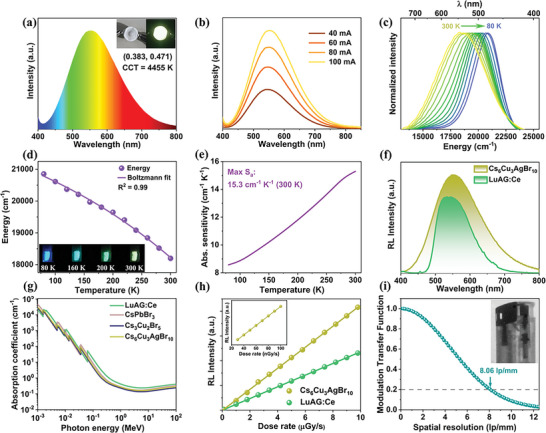
a) Electroluminescent spectrum of the WLED based on Cs_6_Cu_3_AgBr_10_. The insets show photographs of the WLED. b) Electroluminescent spectrum of the WLED driven at different driving currents. c) Temperature‐dependent emission spectra (λ_ex_ = 300 nm) of Cs_6_Cu_3_AgBr_10_ over the 80−300 K range. d) Temperature dependence of emission peak energy. Purple line stands for Boltzmann function used to fit experimental data. The inset shows the photographs of Cs_6_Cu_3_AgBr_10_ at different temperatures under UV irradiation. e) Absolute sensitivity obtained by calculating the first derivative of fitted function. f) RL spectra of Cs_6_Cu_3_AgBr_10_ and LuAG: Ce. g) Absorption coefficients of LuAG: Ce, CsPbBr_3_, Cs_3_Cu_2_Br_5_ and Cs_6_Cu_3_AgBr_10_ as a function of photon energy. h) RL emission intensity of Cs_6_Cu_3_AgBr_10_ and LuAG: Ce as a linear function to dose rate. The inset is the data of Cs_6_Cu_3_AgBr_10_ below 100 nGys^−1^ for detection limit measurement. i) MTF of the flexible film. The spatial resolution of Cs_6_Cu_3_AgBr_10_ is 8.06 lp mm^−1^@ MTF = 0.2. The inset shows the X‐ray image of a lighter imaged by the Cs_6_Cu_3_AgBr_10_‐based scintillator film.

The superior emission performance and high structural stability of Cs_6_Cu_3_AgBr_10_ pave the way for its application in WLED. Therefore, we fabricated a WLED by coating Cs_6_Cu_3_AgBr_10_ on a 365 nm UV LED chip, and the electroluminescence spectrum is shown in **Figure** **5**a. The corresponding Commission Internationale de L'Eclairage (CIE) coordinate is (0.383 and 0.471) and the correlated color temperature (CCT) value is 4455 K, corresponding to warm‐white light (Figure 5a). Furthermore, the as‐fabricated WLED shows high color stability under different driving currents (Figure 5b). These results suggest that Cs_6_Cu_3_AgBr_10_ is a promising candidate for single‐component solid‐state lighting applications. In addition, considering that the film processability of materials is crucial in optoelectronic applications, we used dimethyl sulfoxide (DMSO) to dissolve Cs_6_Cu_3_AgBr_10_ single crystals, and then spin coating the precursor solution on the glass substrate to prepare uniform films. The as‐prepared Cs_6_Cu_3_AgBr_10_ film shows homogeneous warm‐white light consistent with that of single crystals (Figure S17, Supporting Information), which will lay a solid foundation for its assembly in electroluminescent devices.

In addition to lighting, the temperature‐dependent emission property of Cs_6_Cu_3_AgBr_10_ (Figure 4b) makes it promising to act as a luminescent thermometer. When the temperature rises from 80 to 300 K, the emission maximum is shifted from 20 854 to 18 197 cm^−1^ (*ΔE* = 2657 cm^−1^, Figure 5c). Correspondingly, the PL color of Cs_6_Cu_3_AgBr_10_ gradually changes from blue to cyan, then green, and finally warm white, as shown in the inset in Figure 5d. As far as we know, such a significant temperature‐dependent position shift of emission peak has rarely been observed in metal halides. Excitingly, this interesting optical property can be used for band‐shift‐based luminescent thermometry, which can substantially lower systematic interference (e.g., equipment and experimental conditions) compare to intensity‐based luminescence thermometry methods. The energy shift can be fitted using a Boltzmann function:^[^ ^35^, ^36^ ^]^

(2) $$y=A_{2}+\frac{\left(A_{1}-A_{2}\right)}{1+e^{\left[(x-x_{0})/dx\right]}}$$

Absolute sensitivity *S_a_* can be obtained by calculating the first derivative of equation 2, and the maximum *S_a_* obtained at 300 K is 15.3 cm^−1^ K^−1^ (Figure 5e), which is comparable to the highest performance reported for band‐shift thermometers in the literature (Table S3, Supporting Information).

On the basis of the high PLQY and reabsorption‐free emission, Cs_6_Cu_3_AgBr_10_ may also be a promising candidate for efficient X‐ray scintillator. Under X‐ray irradiation, Cs_6_Cu_3_AgBr_10_ single crystal shows a radioluminescence (RL) spectrum similar to that of PL spectrum (Figure 5f), indicating that the radiative recombination channels are the same under X‐ray and UV excitation. Notably, the RL intensity of Cs_6_Cu_3_AgBr_10_ is significantly higher than that of commercially available cerium‐doped lutetium aluminum garnet (LuAG: Ce) scintillation material at the same dose rate. The absorption spectra of Cs_6_Cu_3_AgBr_10_ together with other scintillators in a wide photon energy range are shown in Figure 5g, where the absorption coefficient of Cs_6_Cu_3_AgBr_10_ toward X‐ray is comparable to that of mainstream CsPbBr_3_. To evaluate the scintillation performance, the RL spectra of Cs_6_Cu_3_AgBr_10_ and LuAG: Ce scintillators were measured at various X‐ray dose rates. Both scintillators showed good linearity to X‐ray dose rate in the wide range of 30 nGy s^−1^ to 9.8 µGy s^−1^ (Figure 5h). The light yield of Cs_6_Cu_3_AgBr_10_ is estimated as 45 046 photons MeV^−1^, which is more than two times higher than that of Cs_3_Cu_2_Br_5_ (Figure S18, Supporting Information), comparable with recently reported lead‐free metal halides and much higher than most lead halide perovskites (Table S4, Supporting Information). The detection limit is derived to be 45.9 nGy s^−1^, which is ≈120 times lower than the requirement of standard X‐ray diagnostics (5.5 µGy s^−1^).^[^ ^37^ ^]^ To demonstrate its application in X‐ray imaging, the flexible Cs_6_Cu_3_AgBr_10_@PDMS film with a large size (2.0 × 2.0 cm^2^) and high uniformity was prepared (Figure S19, Supporting Information), and a lighter was used as a target. As shown in the inset of Figure 5i, the internal structure of the lighter could be clearly observed by X‐ray imaging using Cs_6_Cu_3_AgBr_10_‐based scintillator film. The spatial resolution of the Cs_6_Cu_3_AgBr_10_‐based scintillator film is 8.06 lp mm^−1^ for the modulation transfer function at 0.2, which can meet the requirements of a variety of application scenarios such as baggage security checks and nondestructive flaw detection.^[^ ^38^ ^]^

## 3 Conclusion

In summary, we demonstrate the feasibility of incorporating B‐site heterometal as a structural regulation strategy to achieve highly efficient warm‐white light emission in 1D all‐inorganic Cu‐Ag bimetallic halide. After introducing Ag atoms into 0D Cs_3_Cu_2_Br_5_ to form 1D Cs_6_Cu_3_AgBr_10_, the PL color changes from blue to warm‐white, the excitation band edge shifts from 330 nm to 380 nm, and the PLQY increases from 33.8% to 94.5%. Comprehensive spectroscopic study, along with theoretical calculation, reveal that the weakening of electron‐phonon coupling is the key to boosting the triplet STEs emission. In addition, we confirmed that the introduction of Ag atoms significantly improved the stability of Cs_6_Cu_3_AgBr_10_. The excellent PL properties integrated with remarked stability enable Cs_6_Cu_3_AgBr_10_ to demonstrate high performance in solid‐state lighting, temperature detection, and X‐ray imaging. This work not only validates that structural regulation is an effective approach to tailor the photoelectric properties of lead‐free metal halides, but also provides new perspectives on creating single‐component white‐light emitters for advanced lighting and detection applications.

## Conflict of Interest

The authors declare no conflict of interest.

## Supporting information

Supporting Information

## Data Availability

The data that support the findings of this study are available from the corresponding author upon reasonable request.
