# Supplementary material for: Achieving Highly Efficient Warm‐White Light Emission in All‐Inorganic Copper‐Silver Halides via Structural Regulation
Source: Adv Sci (Weinh). 2023 Aug 3;10(28):2303501. doi: 10.1002/advs.202303501 (PMC10558639; doi:10.1002/advs.202303501)
Supplement: Supplementary file 1 — Supporting Information [file ADVS-10-2303501-s001.pdf]

ADVANCED SCIENCE
Open Access

Supporting Information

for *Adv. Sci.*, DOI 10.1002/advs.202303501

Achieving Highly Efficient Warm-White Light Emission in All-Inorganic Copper-Silver Halides via Structural Regulation

*Sijia Wang, Runze Liu, Juntao Li, Fengke Sun, Qing Yang, Shunshun Li, Jianyong Liu, Junsheng Chen and Pengfei Cheng**

Supporting Information

Achieving Highly Efficient Warm-White Light Emission in All-Inorganic Copper-Silver Halides via Structural Regulation

*Sijia Wang, Runze Liu, Juntao Li, Fengke Sun, Qing Yang, Shunshun Li, Jianyong Liu, Junsheng Chen, and Pengfei Cheng**

Sijia Wang, Fengke Sun, Qing Yang, Shunshun Li, Prof. Jianyong Liu, Prof. Pengfei Cheng

State Key Laboratory of Molecular Reaction Dynamics, Dalian Institute of Chemical Physics, Chinese Academy of Sciences, Dalian 116023, P. R. China

E-mail: pfcheng@dicp.ac.cn

Sijia Wang, Juntao Li, Fengke Sun, Prof. Jianyong Liu, Prof. Pengfei Cheng

University of Chinese Academy of Sciences, Beijing 100039, P. R. China

Runze Liu

Institute of Molecular Sciences and Engineering, Institute of Frontier and Interdisciplinary Science, Shandong University, Qingdao 266237, P. R. China

Juntao Li

Key Laboratory of Chemical Lasers, Dalian Institute of Chemical Physics, Chinese Academy of Sciences, Dalian, Liaoning 116023, P. R. China

Junsheng Chen

Nano-Science Center and Department of Chemistry, University of Copenhagen, Universitetsparken 5, DK-2100 Copenhagen, Denmark

1. Materials and Sample Preparation

1.1 Chemical.

CsBr (99.9%), AgBr (99.9%), CuBr (99%) were purchased from the Beijing InnoChem Science & Technology Co., LTD. Hydrobromic acid (48% w/w aq. soln.) was purchased from the Shanghai Aladdin Bio-Chem Technology Co., LTD. Hypophosphorous acid (50% w/w aq. soln.) was purchased from the Sigma-Aldrich company. All of the chemicals were used as received.

1.2 Preparation of $Cs_6Cu_3AgBr_{10}$ and $Cs_3Cu_2Br_5$ single crystals.

$Cs_6Cu_3AgBr_{10}$ single crystals were prepared by mixing 4 mmol of CsBr, 1 mmol of CuBr and 1 mmol of AgBr in 5 mL of HBr and 1 mL of H_3PO_2 aqueous solution. The solution was stirred at 100 ℃ until the solution turned colorless transparent. The solution was then cooled to room temperature to afford white transparent needle-like crystals. For the $Cs_3Cu_2Br_5$ single crystals for contrast, the same method was adopted except for the 1 mmol of CuBr and 1 mmol of AgBr changing to 2 mmol CuBr. The as-synthesized crystals were then collected by suction filtration and dried in the vacuum oven at 70 ℃ for 24 h.

1.3 Preparation of $Cs_6Cu_3AgBr_{10}$ precursor solution.

0.15 M $Cs_6Cu_3AgBr_{10}$ precursor was prepared by adding 0.15 mmol $Cs_6Cu_3AgBr_{10}$ powder into 1 mL DMSO in a 5 mL vial. The mixture was stirred in a nitrogen-filled glovebox at room temperature for 1 h until completely dissolved. After that, a colorless and transparent solution was obtained.

1.4 Preparation of $Cs_6Cu_3AgBr_{10}$ film.

The $Cs_6Cu_3AgBr_{10}$ thin film was fabricated by an antisolvent drop-casting method. Specifically, 50 μL of precursor solution was spin-coated on top of the quartz plate via two-step process, including a low-speed spinning for a short time (500 rpm, 5 s) and a high-speed spinning for a long time (2000 rpm, 55 s). After 45 s, 120 μL of toluene was dripped quickly onto the spinning layer. After the spin coating, we annealed the film at 70 ℃ for 30 min on a heating plate to obtain a uniform, yellowish-white emission film.

2. Characterization methods and Simulation details

2.1 Single Crystal X-ray Diffraction (SCXRD) Analysis.

A suitable single crystal of $Cs_6Cu_3AgBr_{10}$ was selected under a microscope. SCXRD was performed on the Bruker APEX-II CCD diffractometer. Data were collected using graphite monochromatic Mo Kα radiation (λ = 0.71073 Å) at 120 K. The structure was solved and refined through the SHELXL software package. The accession number for the crystallographic data of $Cs_6Cu_3AgBr_{10}$ in this paper is Cambridge Crystallographic Data Center (CCDC): 2206402.

2.2 Powder X-ray Diffraction (PXRD) Analysis.

PXRD was conducted on the PANalytical Empyrean X-ray diffractometer using Cu Kα radiation (λ = 1.54056 Å) over the Bragg angle range of 10-60° at room temperature. PXRD of $Cs_6Cu_3AgBr_{10}$ was compared with the simulated one, and PXRD of $Cs_3Cu_2Br_5$ was compared with the standard spectrogram on Highscore software loaded with PDF-2004 card library.

2.3 X-ray photoelectron spectroscopy (XPS).

The crystals were ground into powders and pressed into a flat sample sheet. XPS was carried out using the Thermo Scientific ESCALAB 250Xi photoelectron spectrometer using Al Kα radiation (hν = 1486.7 eV).

2.4 Scanning electron microscopy (SEM) and Energy dispersive X-ray spectroscopy (EDS)

Single crystals were glued to conductive tape and then plated with platinum. SEM images and EDX were acquired on the JEOL JSM-7800F microscope equipped with an Oxford X-Max silicon drift detector.

2.5 UV-vis absorption spectra.

The UV-vis absorption spectra were measured on the Shimadzu UV-2600 spectrometer equipped with an integrating sphere over the spectral range of 190-400 nm at room temperature. $BaSO_4$ tablet was used as a blank reference.

2.6 Series of Photoluminescence (PL) and PL excitation (PLE) spectra.

Steady-state PL and PLE spectra were recorded on the Edinburgh FLS1000

fluorescence spectrometer using the Xenon lamp at room temperature. Temperature-dependent PL spectra were performed on the FLS1000 spectrometer in the cryostat mode using liquid nitrogen to cool the samples. Time-resolved PL spectra were measured by time-correlated single photon counting (TCSPC) method using the μF lamp.

2.7 PLQY measurement.

The PLQY of samples was acquired on the Edinburgh FLS1000 fluorescence spectrometer equipped with the integrating sphere.

2.8 Excitation power dependent PL spectra measurement.

The excitation power dependent PL spectra were obtained by using a homemade NTAS LFP1000 system. The sample was excited by 355 nm laser pulses with 6 ns pulse, 3 Hz repetition rate and adjustable power. A 450 W tunable pulsed xenon lamp with window from 360 to 700 nm was used as the probe light source.

2.9 Femtosecond transient absorption measurement.

Femtosecond transient absorption measurements were performed by a pump-probe laser system (800 nm, 35 fs, and 1 kHz repetition rate) based on a regenerative amplified Ti: sapphire laser source (Spectra Physics). The samples were excited by 310 nm laser pulses generated by a TOPAS Optical Parametric Amplifier (OPA) which was pumped by the 800 nm pulse. A white light continuum (WLC) generated by CaF_2 was used as the probe beam.

2.10 Thermogravimetric Analysis (TGA) and Differential Scanning Calorimetry (DSC).

TGAs were performed with the Netzsch STA 449 F3 Jupiter thermo-microbalance at a heating rate of 10 °C/min in the range of 40-1000 ºC, using 9.5658 mg powder in an alumina pan. DSCs were performed by the Netzsch DSC 200 F3 differential scanning calorimeter at a speed of 10 °C/min within -160-20 ºC.

2.11 Simulation details.

All DFT calculations were carried out by the Vienna Ab initio Simulation Package (VASP) suite with the projector augmented wave (PAW) method.[1,2] Geometry

optimizations were calculated based on Perdew−Burke−Ernzerhof (PBE) functional built in the generalized gradient approximation (GGA).[3] A kinetic energy cutoff of 500 eV was employed for all calculations and the Brillouin zone was sampled with 2x3x3 Monkhorst-Pack grid. The relaxation convergence for all atoms was reached until the SCF energy and residual force difference fell below 1×10^{-6} eV and 0.01 eV/Å, respectively. For $Cs_6Cu_3AgBr_{10}$, some positions have a fractional occupancy for Ag/Cu atom. The structure was determined by calculating the energy of all possible equivalent occupation for Ag and Cu, and the energetically most stable structure was selected. The electronic band structure and density of states (DOS) were further calculated by Heyd-Scuseria-Ernzerh (HSE06) hybrid functional in order to have a better description for the band gap values.[4] The VASPKIT code was utilized for the post-processing of the calculation results.[5]

2.12 Down-conversion LEDs preparation and measurement.

The single-component warm-white down-conversion LED was fabricated using a 365 nm UV chip and as-prepared yellowish-white emitter $Cs_6Cu_3AgBr_{10}$ powders mixed with UV light adhesives. The photoelectric properties, including the emission spectra, correlated color temperature (CCT), and CIE color coordinates of LED were measured using the OHSP-350M LED Fast-Scan Spectrophotometer equipped with an integrating sphere.

2.13 Scintillator property measurement.

The radioluminescence (RL) spectra were measured using the Edinburgh FLS1000 fluorescence spectrometer equipped with a Mini-X X-ray tube at an operating voltage of 22 keV. The radiation dose rates were adjusted by changing the X-ray source current. For light yield measurement, we selected LuAG:Ce as the reference with a known light yield of 25000 photon/MeV. Then, the light yield of both samples can be estimated using equation 1:

$$\frac{LY_{Sample}}{LY_{LuAG:Ce}} = \frac{R_{sample}}{R_{LuAG:Ce}} \times \frac{\int I_{LuAG:Ce}(\lambda)S(\lambda)d\lambda / \int I_{LuAG:Ce}(\lambda)d\lambda}{\int I_{Sample}(\lambda)S(\lambda)d\lambda / \int I_{Sample}(\lambda)d\lambda} \qquad (1)$$

The detection limit can be derived as the signal-to noise ratio equal to 3.

For X-ray imaging, the radiation dose rates were calibrated by a highly sensitive X-ray ion chamber dose meter (Radcal Corporation 10 × 5-180). The objects and scintillator wafers were placed vertically to the incident X-rays, and the scintillators were fixed just behind the objects. A reflector was utilized to deflect the optical path by 90° to diminish the negative influence caused by direct radiation from the X-ray source on the camera. To collect X-ray images, a CMOS camera was equipped. The spatial resolution can be determined by the spatial frequency value when MTF = 0.2. The MTF curve was calculated by the slanted-edge method.[6]

3. Supporting Tables and Figures

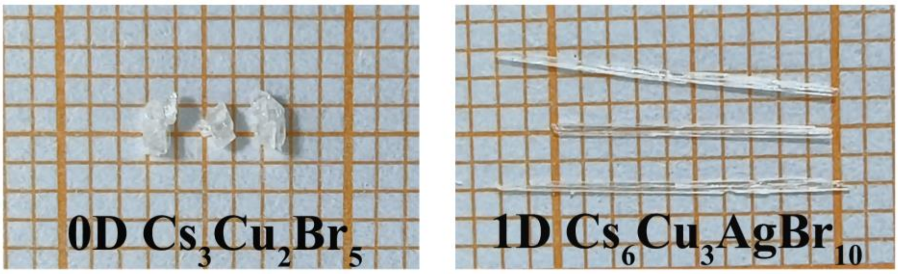

Figure S1. Photographs of as-prepared $Cs_3Cu_2Br_5$ and $Cs_6Cu_3AgBr_{10}$ single crystals.

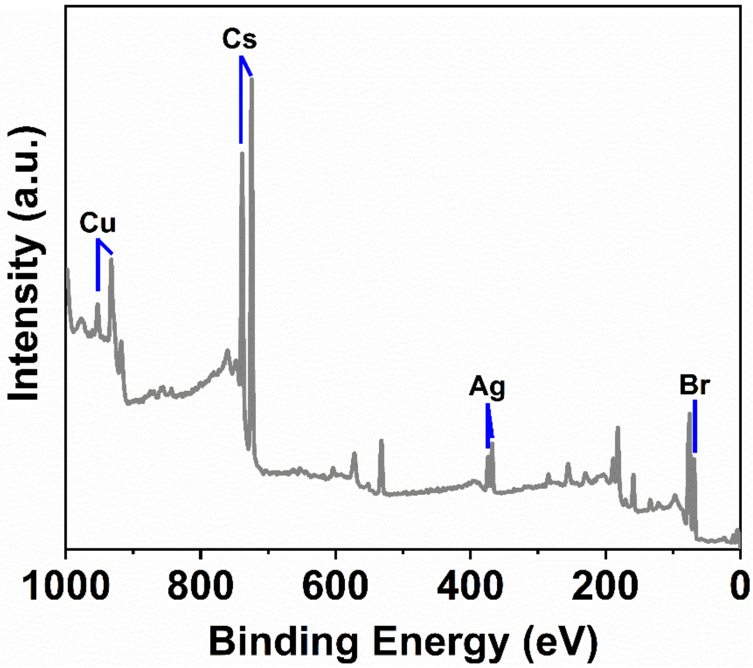

Figure S2. XPS survey spectrum of $Cs_6Cu_3AgBr_{10}$ single crystal.

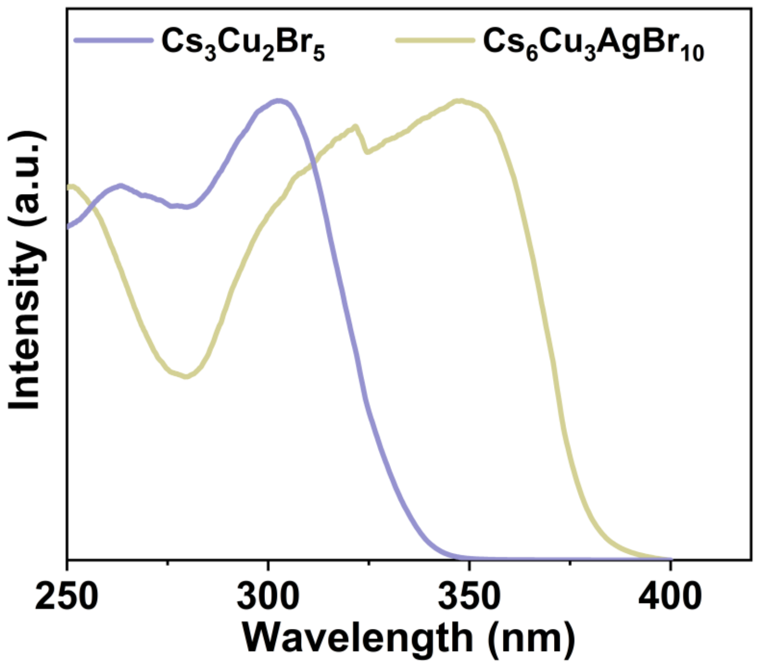

Figure S3. UV-Vis absorption spectra of $Cs_3Cu_2Br_5$ and $Cs_6Cu_3AgBr_{10}$, respectively.

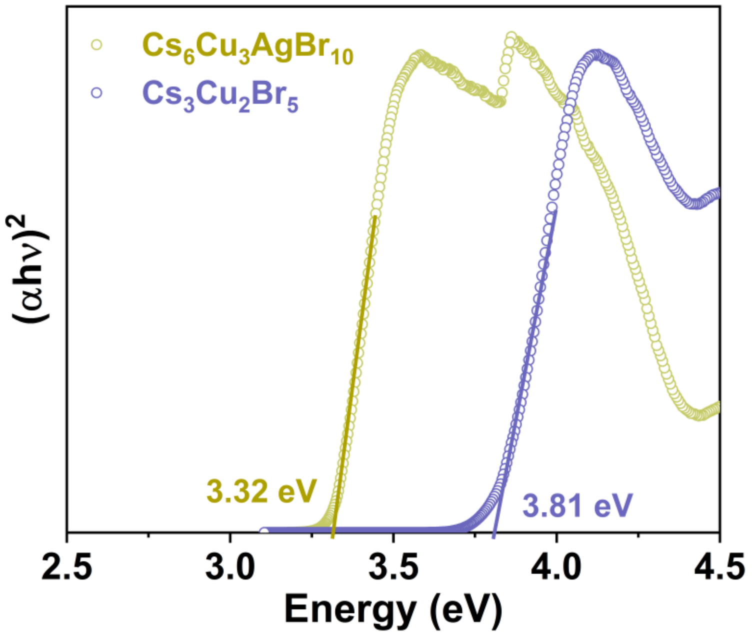

Figure S4. Tauc plots of $Cs_3Cu_2Br_5$ and $Cs_6Cu_3AgBr_{10}$.

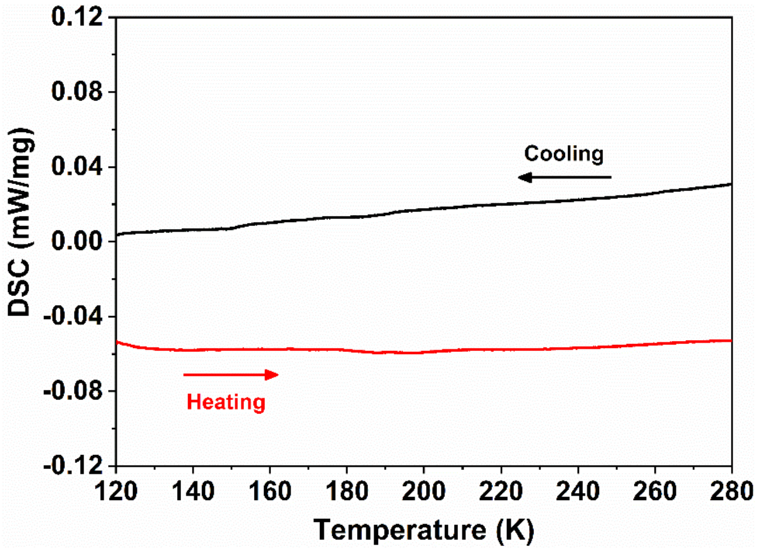

Figure S5. Differential scanning calorimetry (DSC) curves of $Cs_6Cu_3AgBr_{10}$ in a heating/cooling run.

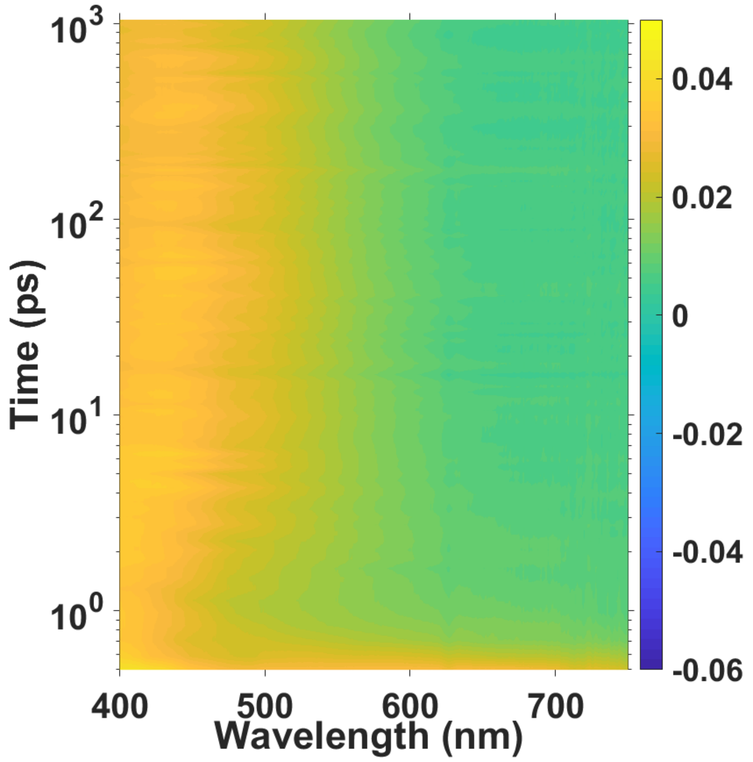

Figure S6. Pseudocolor TA plot of $Cs_3Cu_2Br_5$ upon photoexcitation at 310 nm.

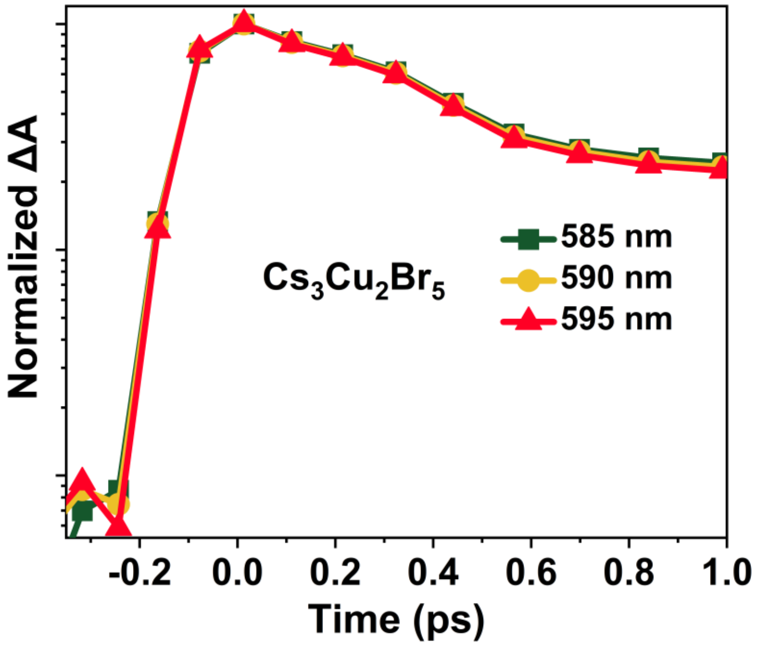

Figure S7. Normalized TA onsets probed at different wavelengths for $Cs_3Cu_2Br_5$.

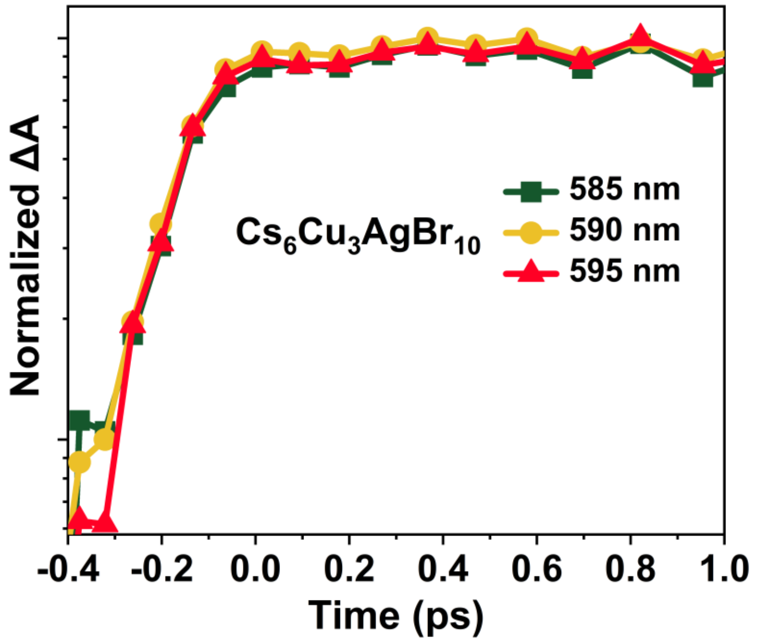

Figure S8. Normalized TA onsets probed at different wavelengths for $Cs_6Cu_3AgBr_{10}$.

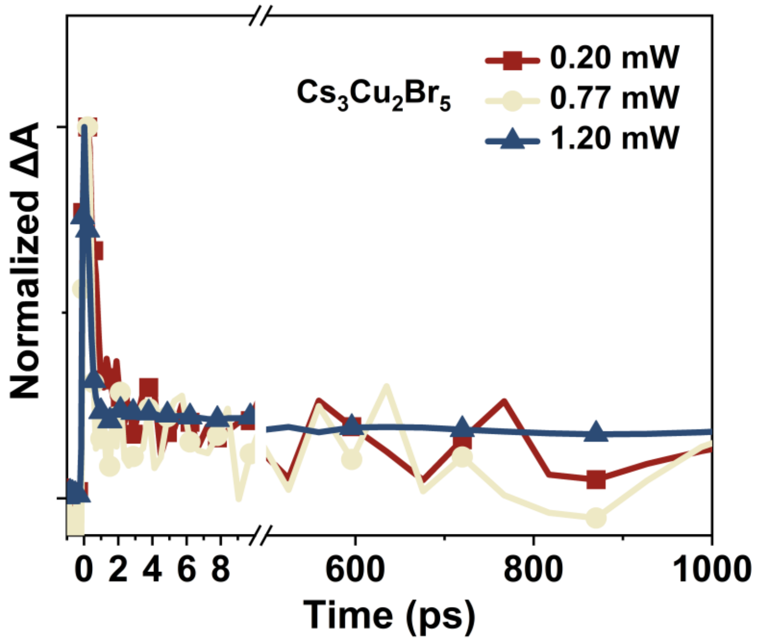

Figure S9. Normalized kinetic decay at 590 nm upon different excitation energies for $Cs_3Cu_2Br_5$. The same decay behaviors under different excitation energies confirm the absence of Auger combination process at present experiment condition.

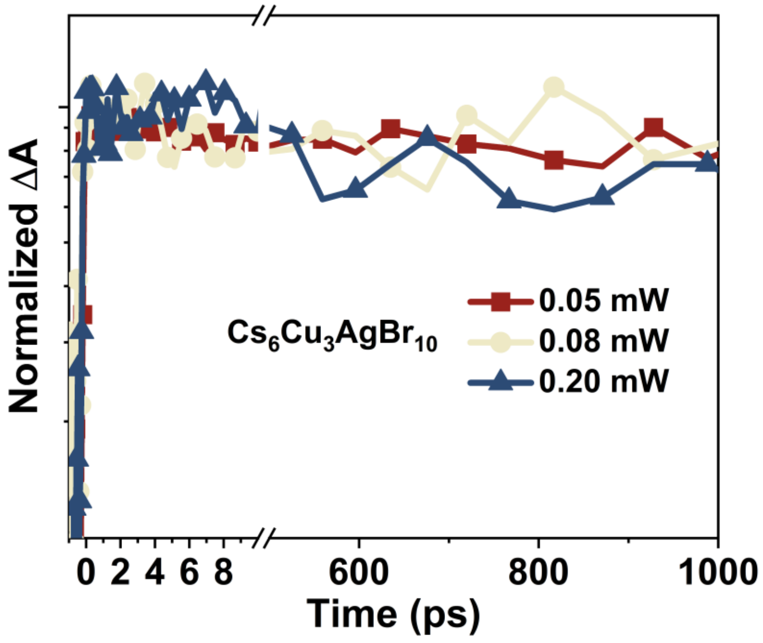

Figure S10. Normalized kinetic decay at 590 nm upon different excitation energies for $Cs_6Cu_3AgBr_{10}$. The same decay behaviors under different excitation energies confirm the absence of Auger combination process at present experiment condition.

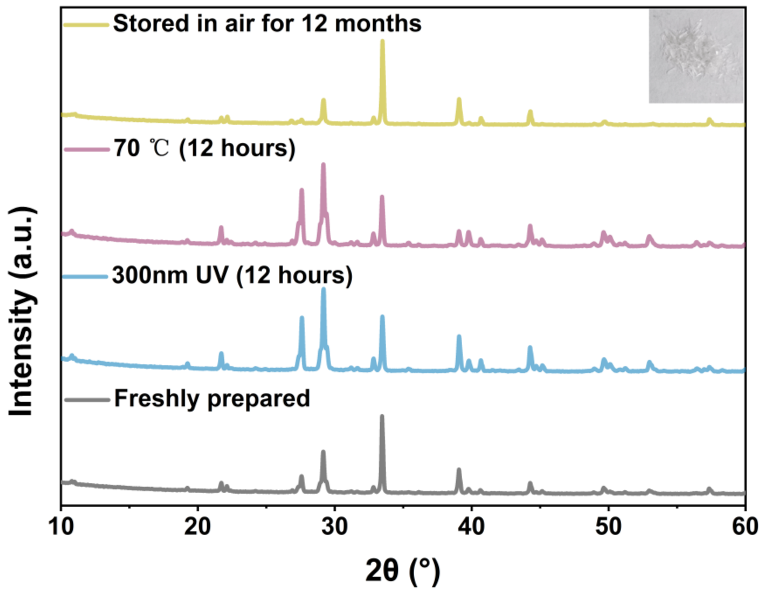

Figure S11. Comparison of XRD patterns of $Cs_6Cu_3AgBr_{10}$ before and after storing in air for 12 months, heating for 12 hours, or 300 nm UV light irradiation for 12 hours. The inset shows the photograph of $Cs_6Cu_3AgBr_{10}$ crystals after being stored in the air for one year.

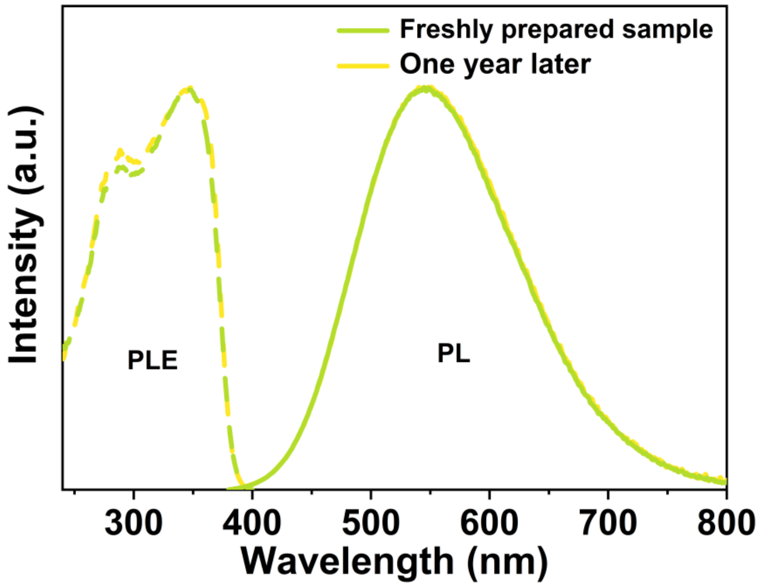

Figure S12. Normalized PLE and PL spectra of the freshly prepared and aged $Cs_6Cu_3AgBr_{10}$ (stored in the ambient atmosphere).

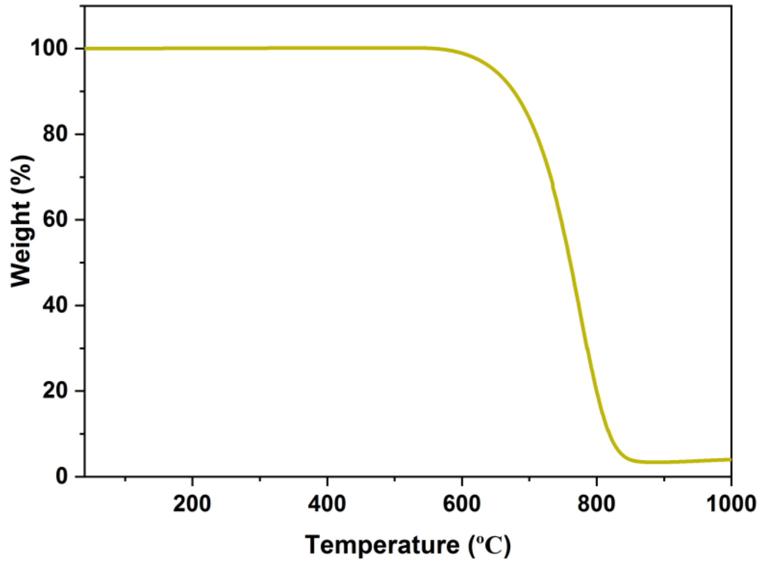

Figure S13. Thermogravimetric analysis (TGA) thermogram of $Cs_6Cu_3AgBr_{10}$.

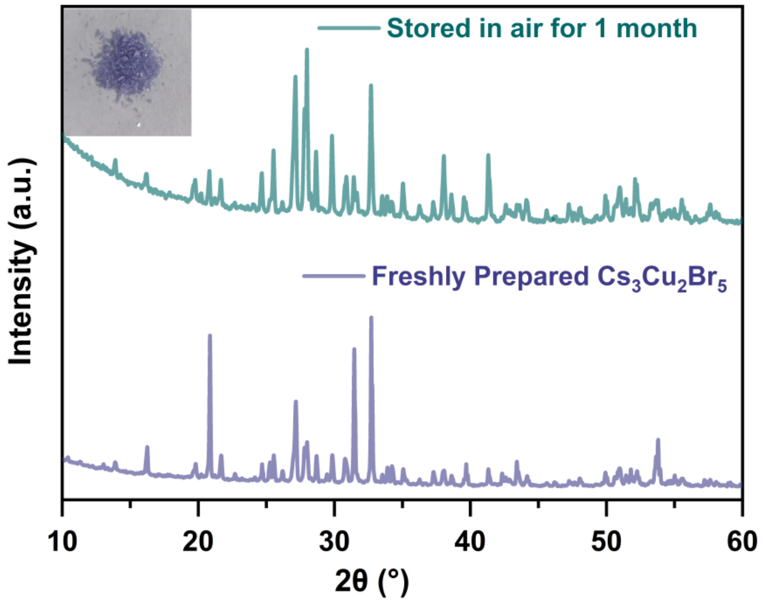

Figure S14. Comparison of XRD patterns of $Cs_3Cu_2Br_5$ before and after storing in air for 1 month. The inset shows the photograph of $Cs_3Cu_2Br_5$ crystals after being stored in the air for one month.

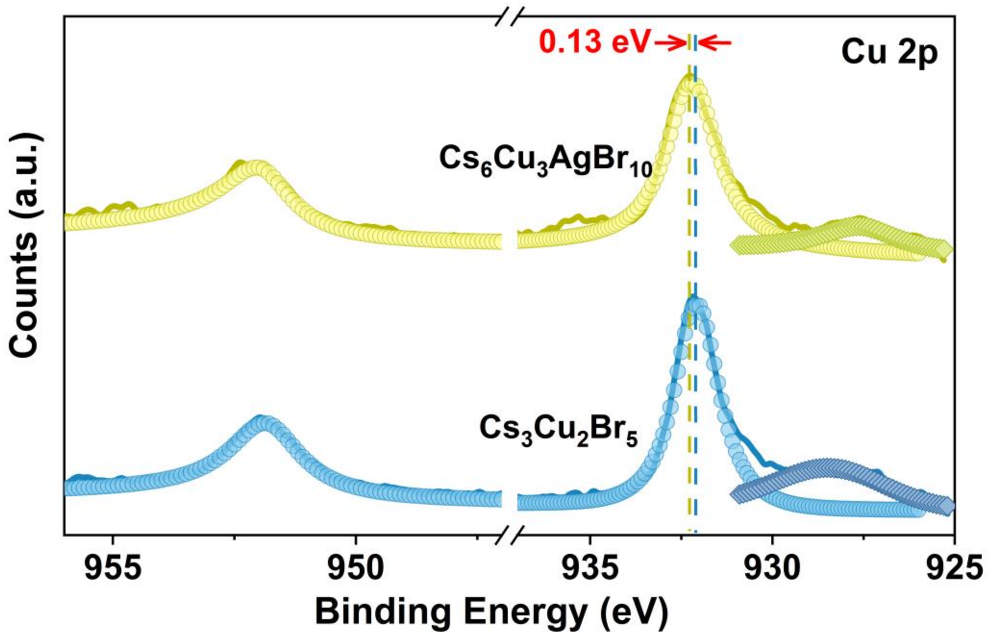

Figure S15. XPS spectra of the Cu 2p regions for $Cs_3Cu_2Br_5$ and $Cs_6Cu_3AgBr_{10}$.

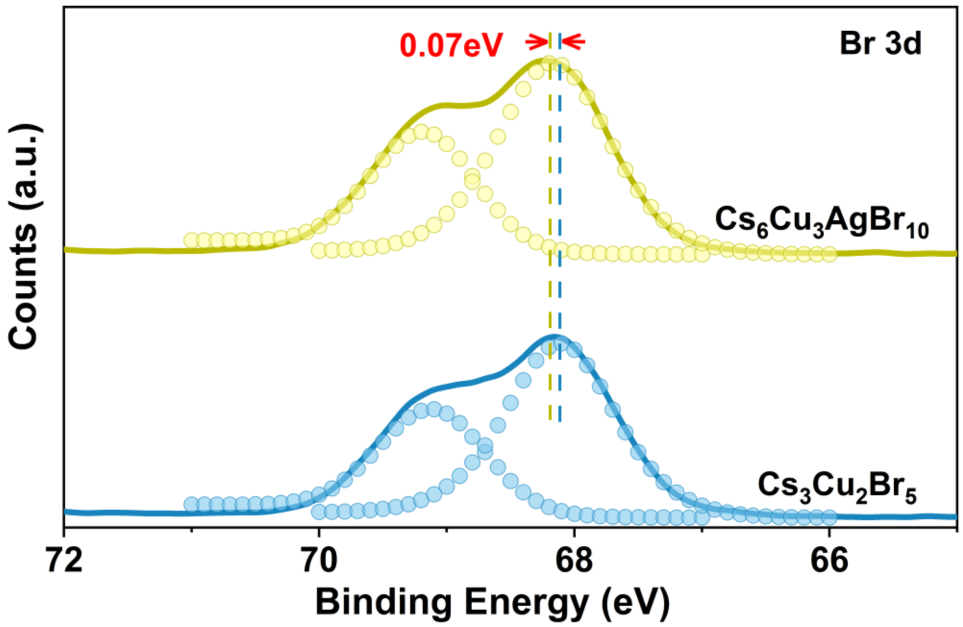

Figure S16. XPS spectra of the Br 3d regions for $Cs_3Cu_2Br_5$ and $Cs_6Cu_3AgBr_{10}$.

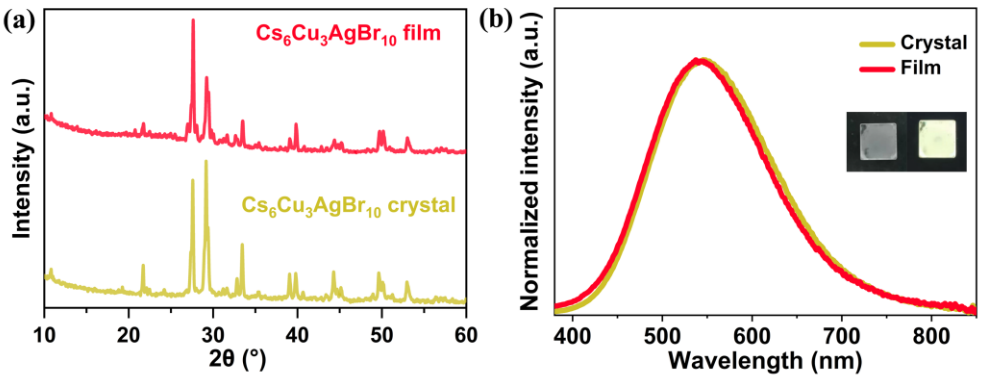

Figure S17. Comparison of (a) powder XRD patterns and (b) PL spectra of $Cs_6Cu_3AgBr_{10}$ crystals and film. The insets show images of the film under ambient light (left) and 300 nm UV light (right) irradiation.

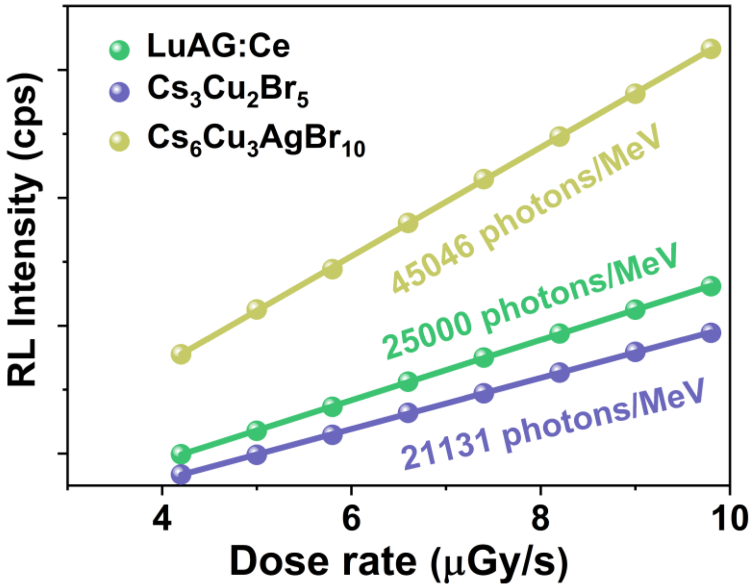

Figure S18. RL emission intensities of $Cs_6Cu_3AgBr_{10}$, $Cs_3Cu_2Br_5$ and LuAG: Ce as a linear function to dose rate.

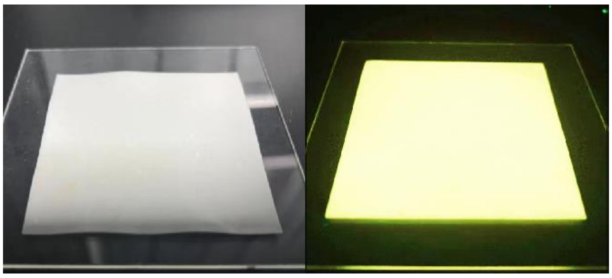

Figure S19. Photographs of $Cs_6Cu_3AgBr_{10}$@PDMS flexible film under ambient condition and under UV excitation.

Table S1. Crystal data and structure refinement information.

Compound	$Cs_6Cu_3AgBr_{10}$
Empirical formula	$Cs_6Cu_3AgBr_{10}$
Formula weight	1895.05
Temperature/K	120
Crystal system	orthorhombic
Space group	Cmcm
a/Å	16.2410(18)
b/Å	9.1301(8)
c/Å	8.9743(7)
α/°	90
β/°	90
γ/°	90
volume/$Å^3$	1330.7(2)
Z	2
ρ_{calc} (g/cm^3)	4.729
μ/mm^{-1}	26.178
F(000)	1628
Crystal size/mm^3	0.2 × 0.15 × 0.1
Radiation	MoKα (λ = 0.71073 Å)
2θ range	5.016 to 55.006
Index ranges	-21 ≤ h ≤ 18, -11 ≤ k ≤ 11, -11≤ l ≤ 11
Reflections collected	4922
Independent reflections	829 [R_{int} = 0.0498, R_{sigma} = 0.0333]
Data/restrain/parameters	829 / 0 / 31
Goodness-of-fit on F^2	1.095
Final R indexes [I>=2σ(I)]	R_1 = 0.0164, wR_2 = 0.0387
Final R indexes [all data]	R_1= 0.0173, wR_2 = 0.0390
Largest diff. peak and hole/ e$Å^{-3}$	0.73 / -0.79

Table S2. Comparison of photoluminescence quantum yields (PLQYs) for recently reported white-light-emitting metal halides.

Compound	PLQY (%)	Refs.
$[(C_3H_7)_4N]_2Cu_2I_4$	91.9	[7]
MA_2CuCl_3	97.0	[8]
$Cs_2Zr_{0.9979}Te_{0.0021}Cl_6$	96.1	[9]
$Cs_6Cu_3AgBr_{10}$	94.5	This work

Table S3. Comparison of absolute sensitivity of different band-shift luminescence thermometers.

Thermometer	S_a (cm^{-1} K^{-1})	Sensing range (K)	Refs.
CdTe quantum dots	5	300–353	[10]
Ag_2S quantum dots	11	295–317	[11]
γ-Al_2O_3:Sm^{2+}	8.0 @ 298 K	298–648	[12]
$[Cp^*Eu(\mu\text{-}BH_4)(THF)_2]_2$	8.2 @ 320 K	60–320	[13]
$[Cp^*Eu(\mu\text{-}BD_4)(THF)_2]_2$	7.07 @ 260 K	30–320	[13]
$[Eu(BH_4)_2(THF)_2]$	5.37@ 220 K	30–320	[13]
$Cs_6Cu_3AgBr_{10}$	15.3 @ 300 K	80–300	This work

Table S4. Comparison of scintillation properties of different X-ray scintillators.

Scintillator	Light yield (photons/MeV)	Detection limit (nGy/s)	Refs.
$CsPbBr_3$	21000	-	[14]
Cs_2ZrCl_6	49400	65	[15]
$Cs_2Ag_{0.6}Na_{0.4}In_{0.85}Bi_{0.15}Cl_6$	39000	19	[16]
$(PPN)_2SbCl_5$	49000	191.4	[17]
$C_4H_{12}NMnCl_3$	50500	36.9	[18]
$(C_8H_{20}N)_2MnBr_4$	24400	24.2	[18]
$Cs_6Cu_3AgBr_{10}$	45046	45.9	This work

References

[1] G. Kresse, J. Furthmüller, *Phys. Rev. B* **1996**, *54*, 11169.

[2] G. Kresse, D. Joubert, *Phys. Rev. B* **1999**, *59*, 1758.

[3] J. P. Perdew, K. Burke, M. Ernzerhof, *Phys. Rev. Lett.* **1996**, *77*, 3865.

[4] J. Heyd, G. E. Scuseria, *J. Chem. Phys.* **2003**, *118*, 8207.

[5] V. Wang, N. Xu, J.-C. Liu, G. Tang, W.-T. Geng, *Comput. Phys. Commun.* **2021**, *267*, 108033.

[6] X. Hu, P. Yan, P. Ran, L. Lu, J. Leng, Y. M. Yang, X. Li, *J. Phys. Chem. Lett.* **2022**, *13*, 2862.

[7] H. Peng, Y. Tian, X. Wang, T. Huang, Z. Yu, Y. Zhao, T. Dong, J. Wang, B. Zou, *ACS Appl. Mater. Interfaces* **2022**, *14*, 12395.

[8] X. Meng, S. Ji, Q. Wang, X. Wang, T. Bai, R. Zhang, B. Yang, Y. Li, Z. Shao, J. Jiang, K. L. Han, F. Liu, *Adv. Sci.* **2022**, *9*, 2203596.

[9] Y. Liu, Y. Wu, Z. Juan, X. Sun, W. Zhang, H. Zeng, X. Li, *Adv. Opt. Mater.* **2021**, *9*, 2100815.

[10] R. Liang, R. Tian, W. Shi, Z. Liu, D. Yan, M. Wei, D. G. Evans, X. Duan, *Chem. Commun.* **2013**, *49*, 969.

[11] Y. Shen, H. D. A. Santos, E. C. Ximendes, J. Lifante, A. Sanz-Portilla, L. Monge, N. Fernández, I. Chaves-Coira, C. Jacinto, C. D. S. Brites, L. D. Carlos, A. Benayas, M. C. Iglesias-de la Cruz, D. Jaque, *Adv. Funct. Mater.* **2020**, *30*, 2002730.

[12] A. Ćirić, S. Stojadinović, Z. Ristić, I. Zeković, S. Kuzman, Ž. Antić, M. D. Dramićanin, *Adv. Mater. Technol.* **2021**, *6*, 2001201.

[13] R. M. Diaz-Rodriguez, D. A. Gálico, D. Chartrand, E. A. Suturina, M. Murugesu, *J. Am. Chem. Soc.* **2022**, *144*, 912.

[14] Y. Zhang, R. Sun, X. Ou, K. Fu, Q. Chen, Y. Ding, L.-J. Xu, L. Liu, Y. Han, A. V. Malko, X. Liu, H. Yang, O. M. Bakr, H. Liu, O. F. Mohammed, *ACS Nano* **2019**, *13*, 2520.

[15] F. Zhang, Y. Zhou, Z. Chen, M. Wang, Z. Ma, X. Chen, M. Jia, D. Wu, J. Xiao, X. Li, Y. Zhang, Z. Shi, C. Shan, *Adv. Mater.* **2022**, *34*, 2204801.

[16] W. Zhu, W. Ma, Y. Su, Z. Chen, X. Chen, Y. Ma, L. Bai, W. Xiao, T. Liu, H. Zhu, X. Liu, H. Liu, X. Liu, Y. M. Yang, *Light: Sci. Appl.* **2020**, *9*, 112.

[17] Q. He, C. Zhou, L. Xu, S. Lee, X. Lin, J. Neu, M. Worku, M. Chaaban, B. Ma, *ACS Mater. Lett.* **2020**, *2*, 633.

[18] T. Jiang, W. Ma, H. Zhang, Y. Tian, G. Lin, W. Xiao, X. Yu, J. Qiu, X. Xu, Y. Yang, D. Ju, *Adv. Funct. Mater.* **2021**, *31*, 2009973.
